# Individual dairy cow identification based on lightweight convolutional neural network

**DOI:** 10.1371/journal.pone.0260510

**Published:** 2021-11-29

**Authors:** Shijun Li, Lili Fu, Yu Sun, Ye Mu, Lin Chen, Ji Li, He Gong

**Affiliations:** 1 College of Electronic and Information Engineering, Wuzhou University, Wuzhou, China; 2 College of Information Technology, Jilin Agricultural University, Changchun, China; 3 Jilin Province Agricultural Internet of Things Technology Collaborative Innovation Center, Changchun, China; 4 Jilin Province Intelligent Environmental Engineering Research Center, Changchun, China; 5 Jilin Province Information Technology and Intelligent Agricultural Engineering Research Center, Changchun, China; Universita degli Studi di Perugia, ITALY

## Abstract

In actual farms, individual livestock identification technology relies on large models with slow recognition speeds, which seriously restricts its practical application. In this study, we use deep learning to recognize the features of individual cows. Alexnet is used as a skeleton network for a lightweight convolutional neural network that can recognise individual cows in images with complex backgrounds. The model is improved for multiple multiscale convolutions of Alexnet using the short-circuit connected BasicBlock to fit the desired values and avoid gradient disappearance or explosion. An improved inception module and attention mechanism are added to extract features at multiple scales to enhance the detection of feature points. In experiments, side-view images of 13 cows were collected. The proposed method achieved 97.95% accuracy in cow identification with a single training time of only 6 s, which is one-sixth that of the original Alexnet. To verify the validity of the model, the dataset and experimental parameters were kept constant and compared with the results of Vgg16, Resnet50, Mobilnet V2 and GoogLenet. The proposed model ensured high accuracy while having the smallest parameter size of 6.51 MB, which is 1.3 times less than that of the Mobilnet V2 network, which is famous for its light weight. This method overcomes the defects of traditional methods, which require artificial extraction of features, are often not robust enough, have slow recognition speeds, and require large numbers of parameters in the recognition model. The proposed method works with images with complex backgrounds, making it suitable for actual farming environments. It also provides a reference for the identification of individual cows in images with complex backgrounds.

## Introduction

Livestock farming is increasing in scale, informatization and refinement [1]. To achieve automated and informative daily management of large-scale cattle farms, individuals need to be able to be identified [2]. Traditionally, individual identification of cattle has been done by methods that cause permanent damage, such as engraved ear branding, ear tagging and radio frequency identification (RFID) tagging [3]. The ear carving method involves cutting openings in the ears, which is painful and time-consuming and can cause stress or even lead to violent death in severe cases [4]. Ear tags are often lost or damaged. Fosgate et al. [5] found that only 21% of range buffalo could be identified two years after ear tags were applied due to tag losses; therefore, tags are only a short-term solution. RFID technology uses radio waves for target identification and tracking and has become popular in dairy farms [6]. However, RFID systems have some security risks as they consist of tags, transponders and terminal servers and are prone to security problems such as tampering with tag contents, system crashes, and intrusion attacks on servers [7, 8]. At the same time, in the links that need real-time response, such as livestock production and disease prevention, it is difficult to apply this method to the actual cattle farm.

In recent years, there has been a trend towards using machine vision techniques to supervise the identification of cows, which has been led by rapid advances in deep learning [9]. Machine vision technology can achieve intelligent and accurate farming, and has the advantages of being low cost and reducing manual labour requirements. It is also non-contact and does not need to touch the animal for identification, so does not cause stress and can provide continuous long-term monitoring [10]. Many scholars have applied deep learning to cows [11, 12]. Zhao et al. [13] collected side-view videos of cows walking in a straight line to study and evaluate image processing techniques, including four feature extraction methods and two matching methods. The highest recognition accuracy of 96.72% was achieved when the FAST, SIFT and FLANN methods were used for feature extraction, description and matching, respectively. Zhang et al. [14] verified that their proposed deep convolutional network outperformed two traditional models, SIFT [15] and BOF [16], in recognizing individual cows in a farm environment. They conducted multiple comparative experiments with different network layers, convolutional kernel sizes and numbers of nodes in the fully connected layer. Shen et al. [17] used the YOLO model to detect cow targets in a series of side views of cows. They classified each cow by fine-tuning a convolutional neural network model and achieved 96.65% accuracy in individual cow identification. The studies above have used fixed side-view images of cows to train their network models; however, in real farms, cows are in constant motion and twisting of their bodies causes a certain degree of deformation of their side spots, which can influence identification and must be considered in practical applications.

Patrizia Tassinari et al. [18] used the yolov3 algorithm to identify cows moving in a cow pen. The cows’ rumps were used as the main detection area, and an average detection accuracy of 0.64–0.66 was obtained. Li et al. [19] proposed a convolutional neural network-based method for automated and accurate recognition of individual cows. It uses a residual learning inverse convolutional network to denoise cow images to obtain a training dataset. It improves on the InceptionV3 network to serve as a training master network and identifies individual cows from the patterns on their tails. Brahim Achour et al. [20] developed a non-invasive system based entirely on image analysis to identify individual cows and their behaviours based on the patterns on their heads. However, the small area of the head limits the available feature points, which has an impact on the final recognition results. Researchers such as Fumio Okura and Ran Bezen [21, 22] have used RGB-D camera 3D video analysis of cows for target detection and individual identification; however, the accuracy has much room for improvement. Although deep learning is widely used in the field of dairy cattle, the automated monitoring of individual cows is still in an early stage of research. Although some success has been achieved, many techniques cannot be applied to dairy farming in a generalized way [23].

In this study, we built a lightweight convolutional neural network model using the Alexnet model as a skeleton network. We trained the model with our own dataset obtained from a cattle farm, where cows were photographed under different lighting and pollution conditions against complex backgrounds, which are challenging to machine vision. In this paper, we demonstrate the effectiveness of our model on complex and variable datasets and provide a systematic analysis of its various modules.

The rest of the paper is organized as follows: Section 2 outlines the data acquisition and image sample expansion, and provides a background to the experimental method. Section 3 gives a detailed description of how the model was built and improved, then the experimental results are analysed in Section 4. Section 5 discusses the results and their limitations, and makes recommendations for future work.

## Materials and methods

### Image acquisition and expansion

**Fig 1:**
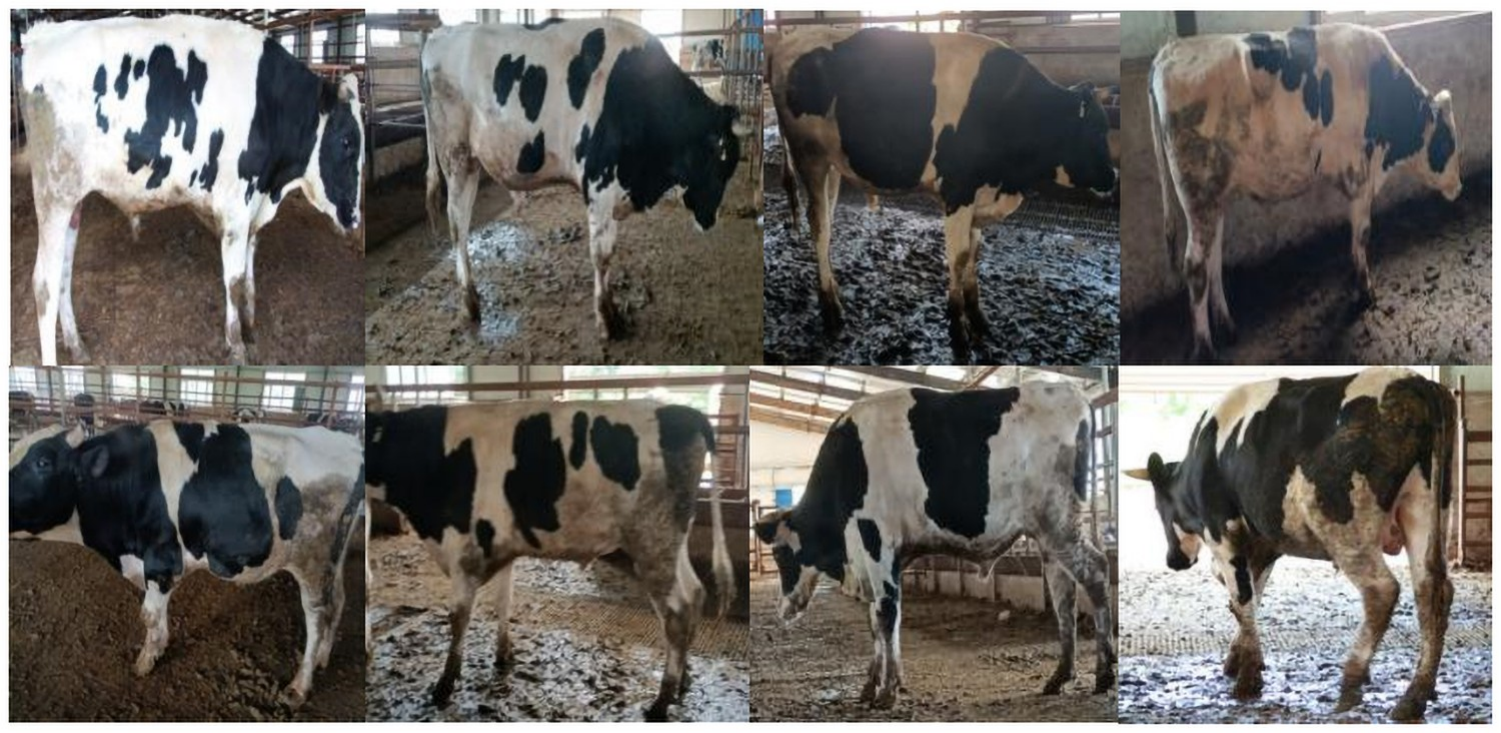
Examples of individual cow images.

In this research, pictures of cows taken against complex backgrounds at a real cattle farm were used for individual recognition. The recognition results of various recognition algorithms are analysed and compared. Lateral view images of Holstein cows were collected at Dongfeng Cattle Farm, Liaoyuan City, Jilin Province, in June 2021, with a Canon EOS 5D Mark II camera with a maximum resolution of 5616 × 3744 pixels. A total of 3772 original images of 13 cows were collected, with some examples shown in Fig 1.

The scale of the dataset has a great impact on the performance of the training network. When the feature space dimension of the samples is larger than the number of training samples, the model is prone to overfitting [24]. To enhance the robustness and generalization ability of the network, the original images were screened to eliminate similar images. Some 1485 sample images were obtained and automatically randomly divided into training and validation sets at a ratio of 8:2 using a Python scripting algorithm. Usually, model training requires a sufficient sample size to avoid overfitting of the model; therefore, the training set was expanded [25]. The traditional ways of expanding a dataset are image flipping, random cropping and colour dithering. In this study, the image rotation method was chosen to expand the training set. The images were randomly flipped from three angles, which tripled the sample size of the training set.

### Experimental method

#### Convolutional neural networks

**Fig 2:**
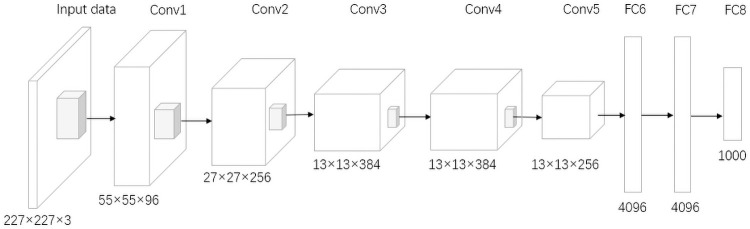
Alexnet model structure.

Traditional machine learning algorithms such as Support Vector Machine (SVM) and k-nearest neighbor (KNN) require prior feature extraction of images. Manual extraction of features is not robust and changes in light and the cows’ physique can lead to feature extraction errors and, thus, poor recognition results. CNNs take the original image as input directly without complicated image preprocessing. Unlike a fully connected feedforward neural network, the neurons in the convolutional layer of a CNN are only connected to the neurons in its adjacent layers. The local connectivity of a CNN can improve the stability and generalization of the network structure, avoid overfitting problems, reduce the total number of weight parameters, accelerate network training, and reduce the memory overhead during computation. Fig 2 shows the classic Alexnet structure, which includes five convolutional layers, three pooling layers, and three fully-connected layers with local connectivity, weight sharing, and pooling [26]. It is relatively simple but it has a large number of weights, leading to long training times, high computational effort, and large storage space requirements, which are not favourable to deployment in arithmetic-constrained environments.

#### Attentional module

**Fig 3:**
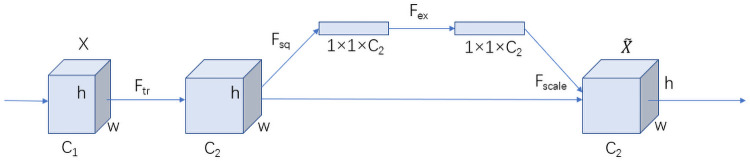
SE block.

By assigning weights to different dimensions, neurons can focus on learning certain regions. Specifically, a superposition step is added to the reasoning process of the model. The data are forcibly converted into weight parameters and superimposed with the original data in different ways. Through the assignment of weights, different regions obtain different levels of importance to make the model quickly locate and focus on identifying these high-weight regions. For a small parameter model, there is no more computing space available to focus on the recognition of cow texture. In order to make more efficient use of these parameters, we introduce an attention mechanism. The attention mechanism we use comes from the Squeeze-and-Excitation Network (SENet), which was the champion model used in the classification task of the last Imagenet large-scale visual recognition challenge [27]. SENet focuses on the importance of linking various channels and learning their characteristics through the network. The SENet module can be divided into two steps: *squeeze* and *excitation*. Squeeze obtains the global compressed feature quantity of the current feature map by performing global average pooling on the feature map layer. Excitation obtains the weights of each channel in the feature map through a two-layer fully connected bottleneck structure and uses the weighted feature map as input to the next layer of the network. Squeeze and excitation are the core procedures of SENet. Its module is shown in Fig 3.

## Model building

### Dairy cow identification process

**Fig 4:**
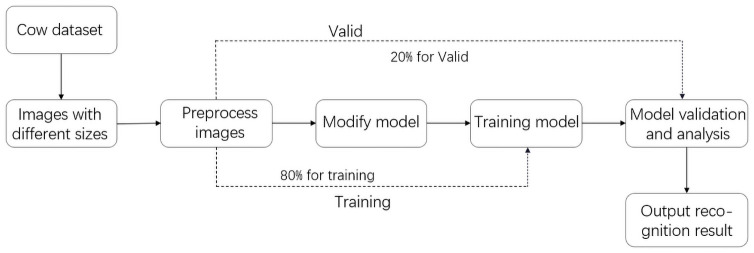
Flowchart of the dairy cow identification process.

The identification process used in this paper is shown in Fig 4. After preprocessing of the acquired image by the method described in Section 2.1, the input images were size-normalized. Then, the dataset was divided into training and validation sets at a ratio of 8:2. We used Alexnet as the skeleton network to build the individual cow recognition model. We used the training set to train the model and the validation set to verify the accuracy and robustness of the model in achieving rapid and effective identification of individual cows.

### Improvements

**Fig 5:**
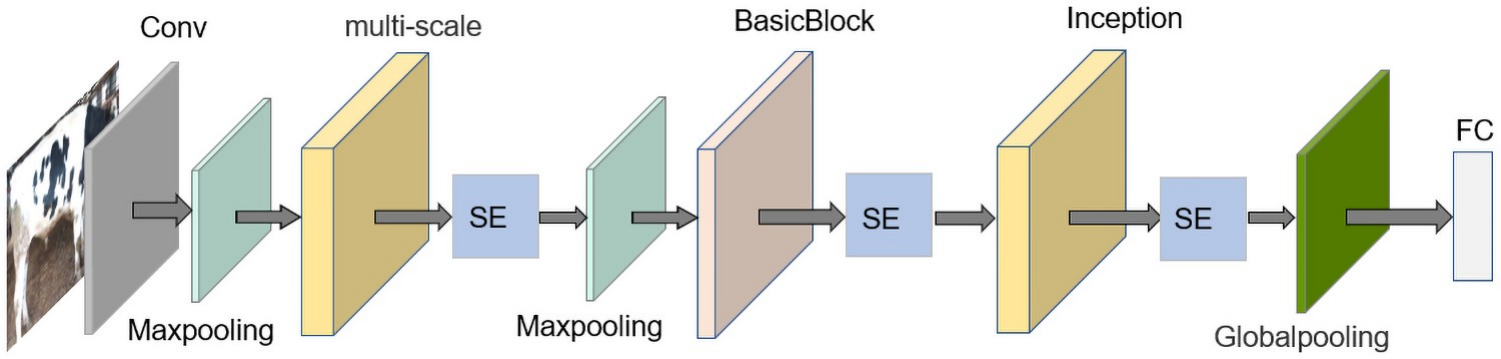
Structure of the model used in this research.

In the Alexnet model, 11 × 11, 5 × 5, and 3 × 3 convolution kernels are mainly used for the extraction of multiscale features. Serial feature extraction in the same dimension is too clumsy and increases the model computation, while the inception module in the GoogLenet network is more reasonable for multiscale feature extraction. Therefore, we only keep part of the convolutional layers of Alexnet, change the convolutional layers into inception modules and add the SE module. We also add the BasicBlock of short-circuit connection to make the feature information circulate better, and replace the fully connected layers with global pooling to ensure that the features are not lost while greatly reducing the number of model parameters. The lightweight convolutional neural network model constructed in this research is shown in Fig 5.

**Fig 6:**
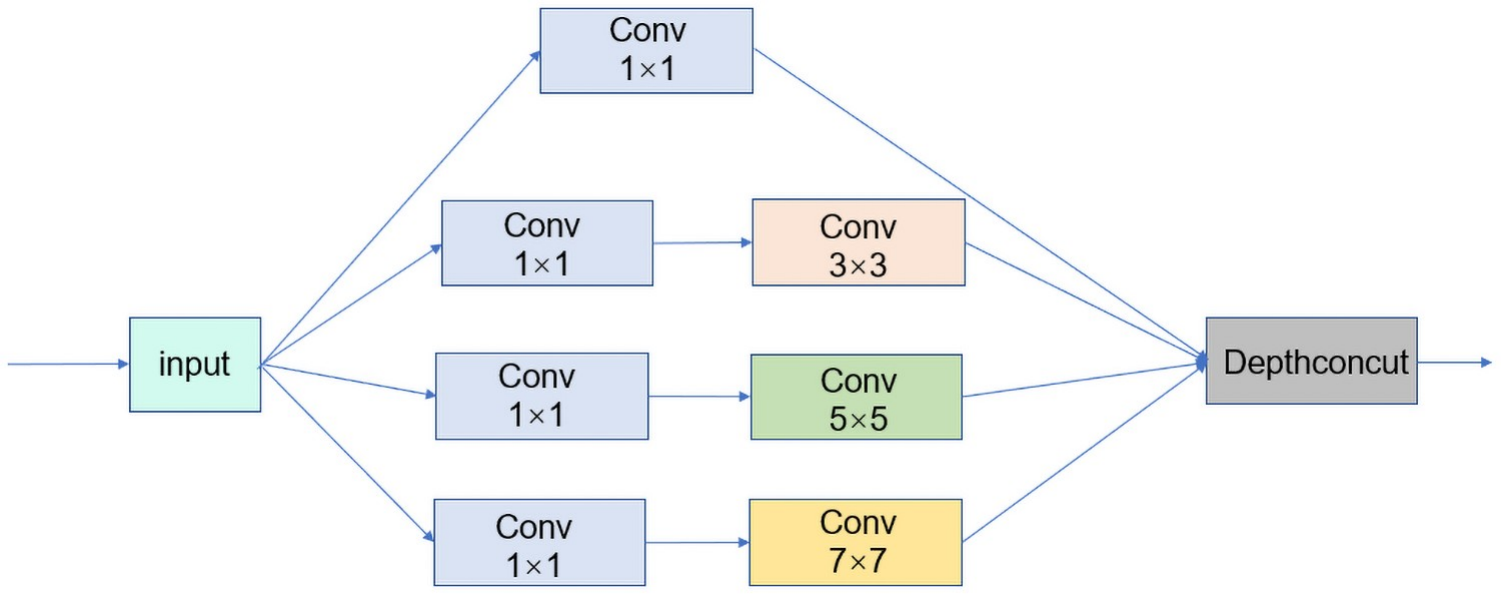
Multi-scale module.

Based on Alexnet, we keep Conv1, which is a convolutional block using an 11 × 11-size convolutional kernel, because the input cow images have many pixels and using this size of kernel accelerates the shrinkage of features. The multi-scale module is added after Conv1, as shown in Fig 6, to reduce the number of overlapping convolutional kernels without affecting the accuracy of the final result. In the multi-scale module, we add a 7 × 7 convolutional kernel to perform multiscale extraction of features to ensure that features are not lost. The improved multi-scale module has four parallel convolution kernels of 7 × 7, 5 × 5, 3 × 3, and 1 × 1, and the *depthconcut* operation is used to merge the feature maps of these parallel outputs. In order to further shrink the extracted effective feature area, we add the SE module after the multi-scale module. This compresses the features into a 1 × 1 size for input to the two fully connected layers, thus assigning weights to different channels of the features so that the model has more nonlinearities and can better fit the complex correlations between channels.

**Fig 7:**
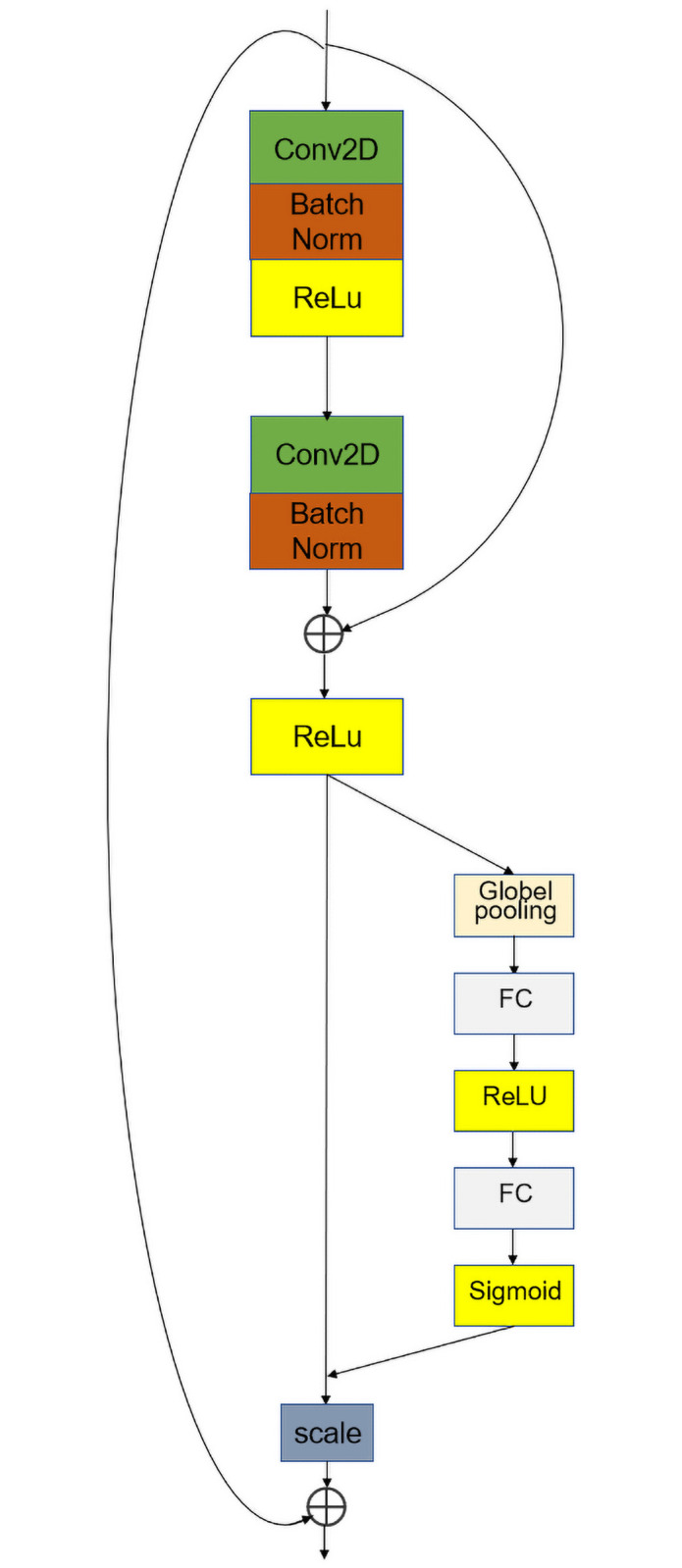
BasicBlock + SE module.

We added the BasicBlock module with short-circuit connections in the middle of this model, as shown in Fig 7, which consists of two 3 × 3 convolutional layers combined. This is responsible for connecting the upper and lower parts of the network and fusing the feature maps output by the multi-channel multi-scale module and does not change the number of channels. The reason why we did not add connections where the dimensions are too high or too low is that the gap between the feature information in different dimensions is too large, and short-circuiting the connections may lead to information in the feature maps after the weights have already been assigned through the attention mechanism confusion occurs. We then added an inception module and an attention mechanism after the block, so that the structure can increase the computational power of the model. If the computational power is too low, the computational performance of the whole network will be too low, thus reducing the accuracy of the model. The model’s computational speed and accuracy are traded off to ensure the highest acceptable performance without increasing the number of parameters.

Finally, we optimize the last three fully connected layers into one global pooling layer and one fully connected layer. The globally pooled feature map aggregates the information more effectively and reduces the computational burden for the fully connected layers. In addition, all convolutional layers are followed by a Batch Normalization (BN)layer to accelerate the convergence of the network and reduce the occurrence of overfitting.

## Results and discussion

### Experimental environment and parameter settings

The deep learning framework used in this experiment was Pytorch 1.8.0. The version of Torchvision was 2.2.4 and the computer configuration was an Intel Core i7-8700 CPU running at 3.20 GHz. It was equipped with an Nvidia GeForce GTX 1080 graphics card. The adapter was an Intel UHD Graphics 630. The software was CUDA API version 0.9.10, based on the Python 3.8.3 programming language and integrated with the PyCharm2020development environment.

To better evaluate the differences between the true and predicted values, the batch training method was used to divide the training and testing process into multiple batches. Each batch contained 32 images, so the bathsize was 32 and the number of iterations was set to 40. The loss function used cross-entropy loss, the weight initialization method used Xavier, the initialization bias was 0, and the initial learning rate of the model was set to the model adopts SGD optimizer and uses softmax classifier. The ratio of the training set to test set was 8:2. To obtain a high training speed while maintaining a good classification rate, the input images were uniformly compressed to 224 × 224 pixels using an interpolation algorithm.

### Experimental results and analysis

**Fig 8:**
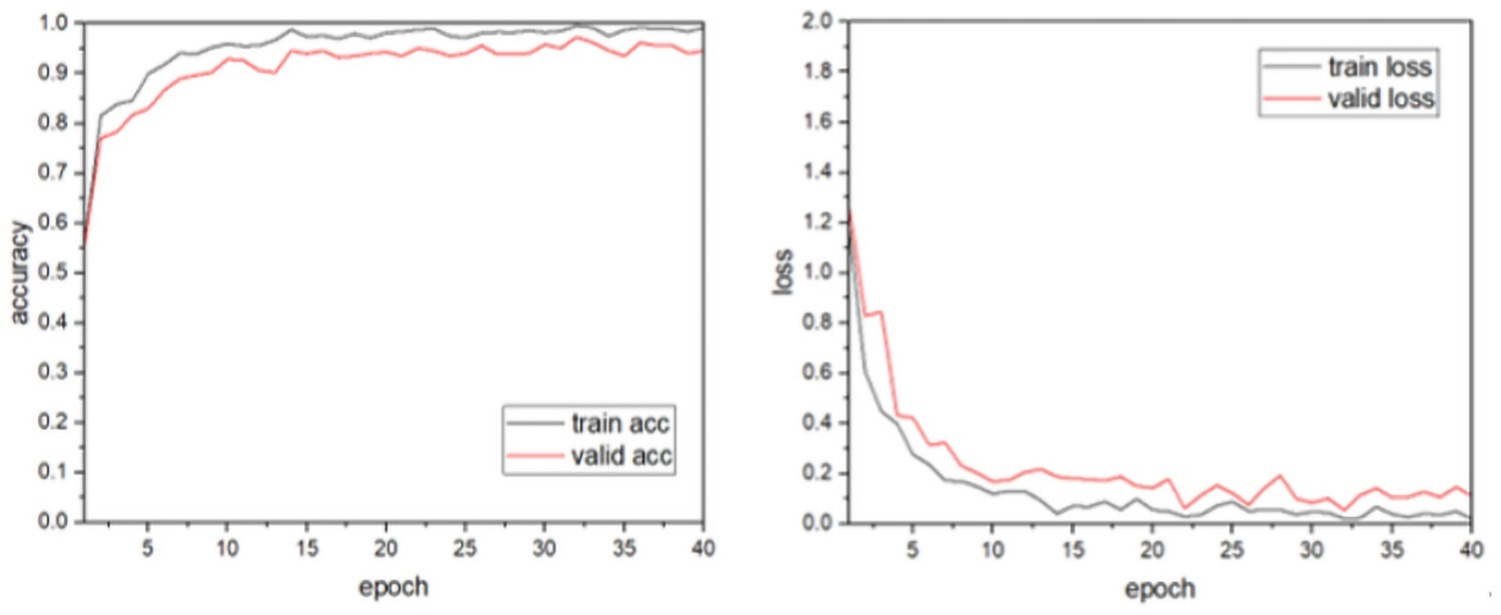
Accuracy of the model trained on the system with the GPU.

The identification of individual cows according to the algorithm and identification process proposed above was explored separately for each of the joined modules, as shown in Table 1. Firstly, the structure of multi-scale+SE was added to improve the accuracy of the model significantly, while the number of parameters and training time were reduced. On this basis, BasicBlock was added to ensure the features were not lost by using the short-circuit connection in the module while extracting the important features to improve the accuracy of the model. The SE module was connected after BasicBlock to decrease the number of parameters; finally, inception+SE was added to complete the model constructions. The final model’s recognition accuracy was 97.95%, which is 10.45% higher than that of the skeleton model (Alexnet network). The number of model parameters was reduced 34-fold and the model size was 6.51 MB. The model we built can achieve 97.95% accuracy in cow identification, with an average loss rate of 0.44%, as shown in Fig 8. The model has fewer parameters and is more applicable to real dairy farm environments.

**Table 1:** Comparative results of module addition.

Improvement method	Accuracy (%)	Total parameters	Parameter size (MB)
Alexnet	87.50	58,314,120	222.45
multi-scale+SE	95.65	3,411,400	13.01
BasicBlock+SE	96.74	2,091,208	7.98
Ours	97.95	1,707,592	6.51

### Model performance validation

**Fig 9:**
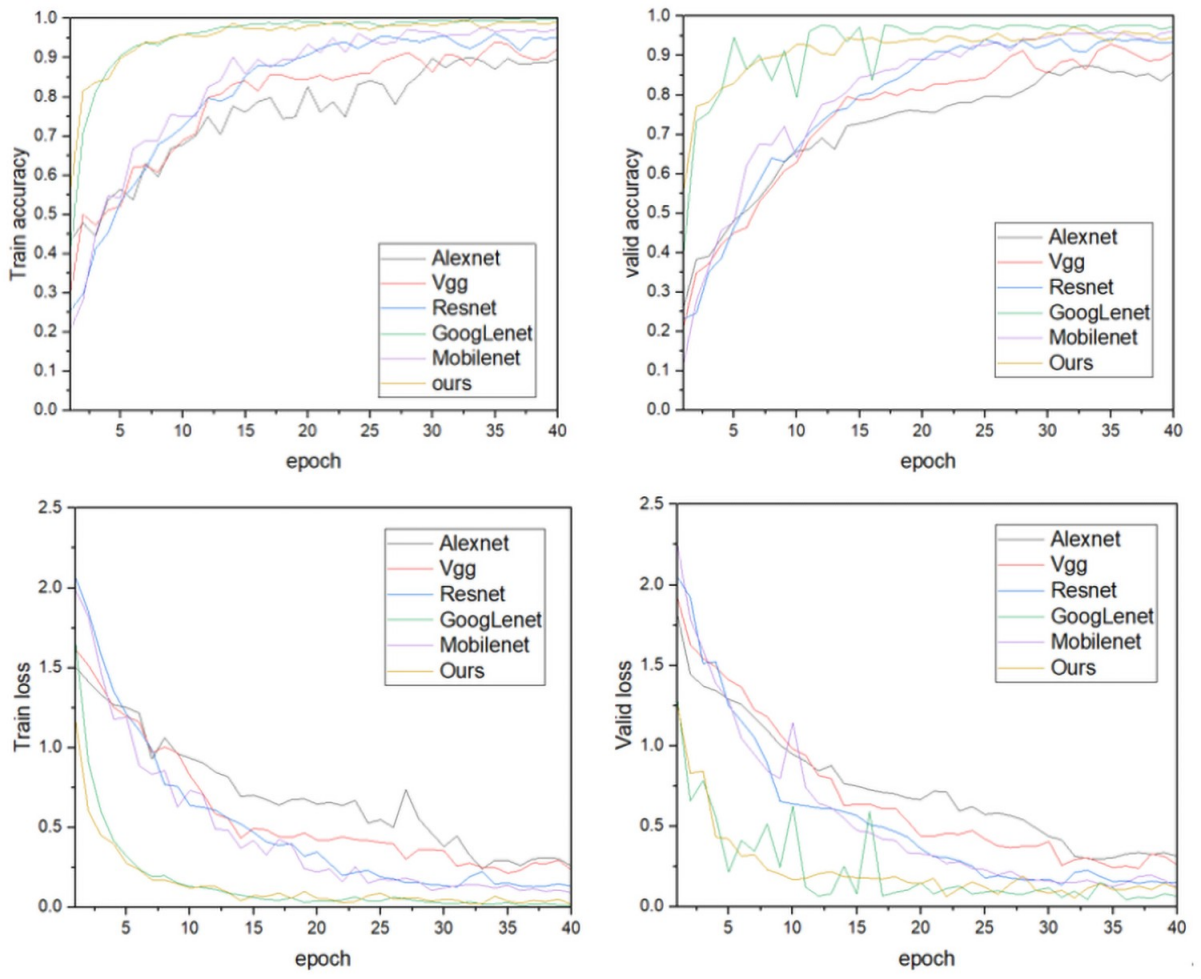
Comparison of the results of different network models.

To verify the effectiveness of the model, it was compared with five other network models. The other convolutional neural network models were trained using cow images with complex backgrounds according to the training parameters and training methods in Section 3.1, and the results are shown in Fig 9. Compared with Alexnet, Vgg16, Resnet50 and Mobilenet v2, our model converges faster and has a high recognition rate of 97.95%. The GoogLenet model is slightly higher in recognition rate but the model is more volatile and unstable in validation, while our model is more robust and has faster convergence and stability in both training and validation. From the comparison of model size and parameter number, as shown in Table 2, the Alexnet, Vgg16 and Resnet50 models have large numbers of parameters and longer average single recognition times with low recognition precision. Hence, they do not allow the rapid identification of individual cows required in actual cattle farms. Although the GoogLenet model was slightly better than our model in terms of recognition accuracy, its parametric size in the training process cannot be ignored. The parameter size was 21.04 MB, which would make it difficult to migrate the model to mobile devices. The final parameter size of Mobilenet v2, which is famous for its light weight, was 8.52, which is 1.3-times that of our model, and its recognition accuracy was lower. It can be seen that the lightweight convolutional neural network model constructed in this study can greatly reduce the number of model parameters while ensuring recognition accuracy. Compared to the other models, it can identify individual cows faster, thereby saving recognition time and breeding costs in actual dairy farms. Hence, it can better meet the application needs of actual complex dairy farm environments.

**Table 2:** Analysis of the results of different models.

Recognition method	Accuracy (%)	Total parameters	Parameter size (MB)	Average single training time (s)
Alexnet	87.50	58,314,120	222.45	34
Vgg16	92.93	134,293,320	512.29	46.5
Resnet50	94.33	23,524,424	89.74	32.45
GoogLeNet	98.61	5,611,179	21.04	21.28
Mobilenet v2	96.20	2,234,120	8.52	21.93
Ours	97.95	1,707,592	6.51	5.75

### Background analysis of the dataset

**Fig 10:**
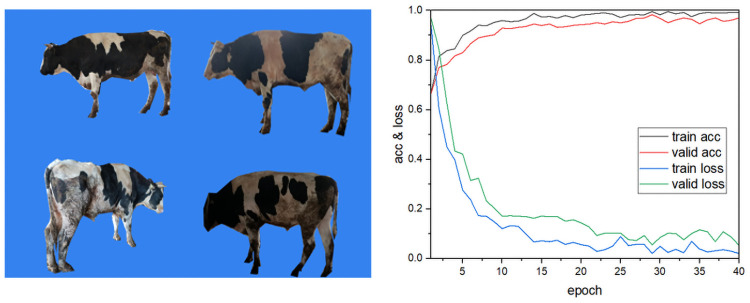
(a) Images of cows with a simple background, (b) accuracy and loss rate curves of the training model with the dataset with simple backgrounds.

**Fig 11:**
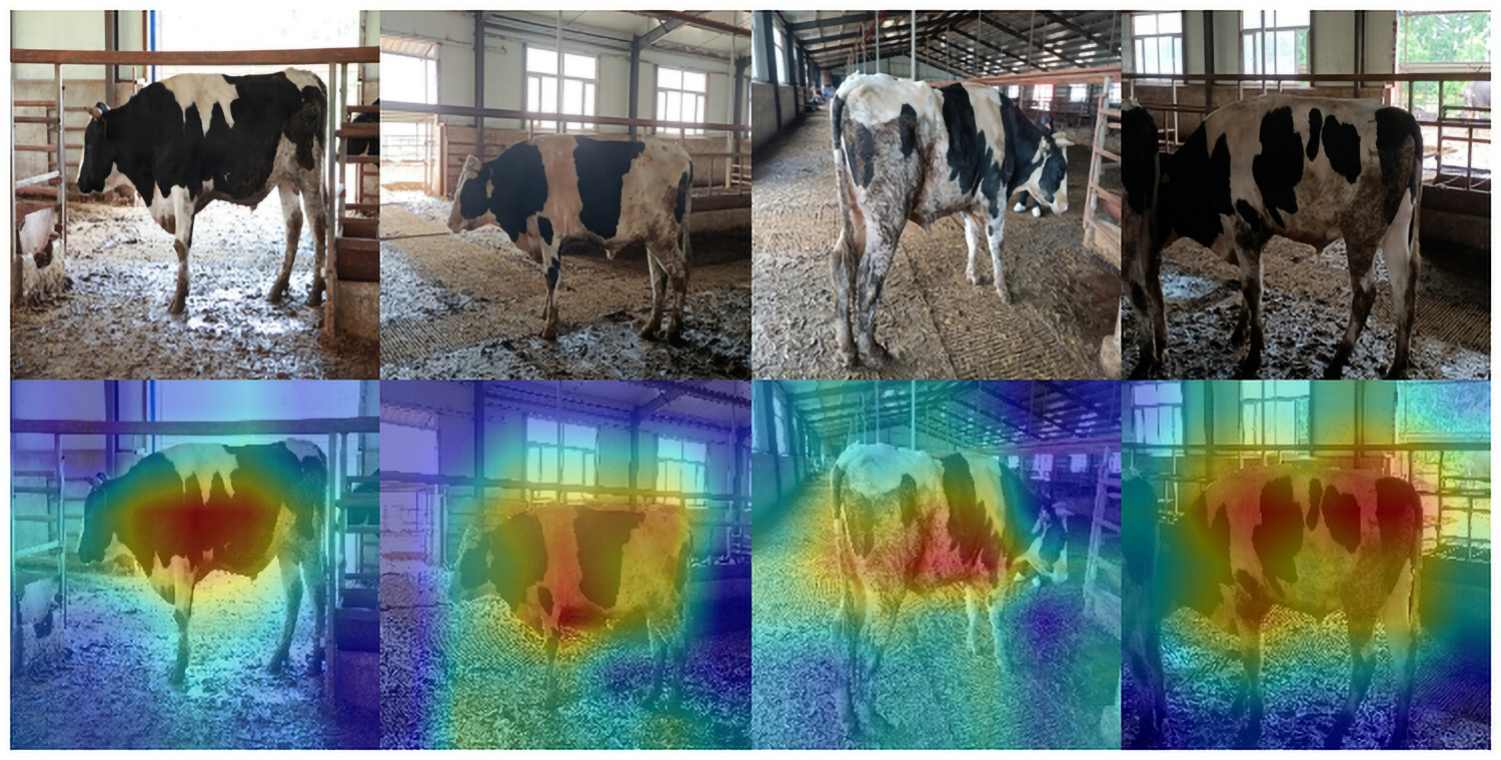
Example cow heat maps.

The data collected in this paper are images of cows with complex backgrounds taken at real cattle farms. To avoid classification bias, we verified whether the proposed model used cows features or backgrounds for recognition. We produced a simple background cow dataset by removing the backgrounds from images of cows with complex backgrounds. Some simple background data are shown in Fig 10(a). We then augmented the image dataset by the data expansion method proposed in Section 2.1, keeping the experimental parameters and training methods constant, and then trained our model in the environment described in Section 4.1. The results are shown in Table 3. The recognition accuracy of the dataset of cow images with complex backgrounds was 97.95%, and that of the dataset with simple backgrounds was 98.32%. The curves of accuracy and loss rate are shown in Fig 10(b). In addition, the image recognition process of the model was visualized; heat maps of cow images after the model recognition process were output, as shown in Fig 11. The results verify the accuracy of the proposed model for cow recognition in images with complex backgrounds.

**Table 3:** Validation of the proposed model with two datasets.

Dataset	Training accuracy (%)	Validation accuracy (%)
Complex	99.27	97.95
Simple	99.58	98.32

## Conclusions

We aimed to improve the accuracy, stability and speed of cow identification. We explored new methods of cow identification with strong practical application capabilities to promote the development of intelligent cattle farming. In this study, we proposed a method for extracting multiscale hierarchical features for cow identification based on a lightweight convolutional neural network. Using a deep learning convolutional neural network, the large network, Alexnet, was used as a skeleton network and was improved by adding a multiscale extraction module. We introduced the short-circuit connection BasicBlock and combined it with the SE attention mechanism to build a lightweight convolutional neural network model to train and recognize the side-view images of cows, achieving a final recognition rate of 97.95%. In comparison with five other networks, our model had the lowest number of parameters, the shortest recognition time, and strongest stability and robustness while ensuring a high recognition rate. In addition, the model was verified using images with simple backgrounds and thermodynamic diagrams. The results show that the model is suitable for identifying cows in complex environments. Our lightweight, high-precision model for identifying individual cows has potential for practical application. Among the models used for cow identification, the proposed model has a small number of parameters; however, there is still room for improvement in recognition accuracy. In future work, while ensuring that the model remains lightweight, we will improve its recognition accuracy. This will provide technical support for individual cow identification in complex environments and provide a scientific basis for intelligent cow breeding management.
