# Correction: Individual dairy cow identification based on lightweight convolutional neural network

**DOI:** 10.1371/journal.pone.0307252

**Published:** 2024-07-11

**Authors:** Shijun Li, Lili Fu, Yu Sun, Ye Mu, Lin Chen, Ji Li, He Gong

An affiliation is missing for the first author. Shijun Li is affiliated with #1 and #2. The correct affiliations are:

**1** College of Information Technology, Jilin Agricultural University, Changchun, China, **2** College of Electronic and Information Engineering, Wuzhou University, Wuzhou, China.
